# Monument in Westminster Abbey to John Hunter

**Published:** 1847-06

**Authors:** 


*Monument in Westminster Abbey to John Hunter.*—Meetings have lately been held at the Royal College of Surgeons, attended by most of the surgeons of the metropolitan hospitals, for the purpose of organizing a committee among themselves, in co-operation with the Council, for the purpose of raising a monument in Westminster Abbey to the memory of John Hunter. The circumstances under which the proposal was made are exceedingly creditable to all parties ; it appears that after the delivery of the late Hunterian oration, when the members of the Council, with their distinguished visiters, dined at the Freemasons' Tavern, the Rev. Dr. Buckland, the eminent geologist, in returning thanks for his health, stated that soon after his installation to the deanery of Westminster, whilst walking round the Abbey with Professor Owen, he expressed to that gentleman his regret that the medical profession, of which Hunter was so bright an ornament, had not yet erected any monument to his memory, and suggested the propriety of one being placed in a vacant niche near Dr. Baillie's, generously offering, should his suggestion be adopted, to forego the fees to which he would have been entitled, and to promote the object as far as he could. The wish thus expressed was immediately responded to by the Council, who have invited the susgeons of the metropolitan hospitals to confer with them on the best means for carrying out the object in question.—*Ibid.*

